# ﻿Clarifying the nomenclature of *Strychnosbredemeyeri* and *Lasiostoma* (Loganiaceae)

**DOI:** 10.3897/phytokeys.243.123921

**Published:** 2024-06-24

**Authors:** Robberson Bernal Setubal, Lena Struwe, Jefferson Prado, Rafaela Campostrini Forzza

**Affiliations:** 1 Departamento de Botânica, Instituto de Biociências, Universidade de São Paulo, Rua do Matão, 277, 05508-090, São Paulo, SP, Brazil; 2 Jardim Botânico do Rio de Janeiro, Rua Pacheco Leão, 915, 22460-030, Rio de Janeiro, RJ, Brazil; 3 Department of Ecology, Evolution, and Natural Resources, Rutgers University, 14 College Farm Road, New Brunswick, NJ 08901, USA; 4 Department of Plant Biology, Rutgers University, 59 Dudley Road, New Brunswick, NJ 08901, USA; 5 Instituto de Pesquisas Ambientais (IPA), Herbário SP, Av. Miguel Estéfano, 3687, CEP 04301-012, São Paulo, SP, Brazil; 6 Instituto Chico Mendes de Conservação da Biodiversidade, Prado, BA, Brazil

**Keywords:** Classification, Gentianales, Neotropics, nomenclature, Strychneae, systematics

## Abstract

*Strychnos* (Loganiaceae, Gentianales) is a large and pantropical genus of woody plants, ethnobotanically important as a source of many toxic alkaloids, including strychnine. Unfortunately, the status of numerous names at various ranks of *Strychnos* remains unresolved, including that of many specific or infraspecific taxa in the Neotropics. In this study, we address *Strychnosbredemeyeri* (basionym *Lasiostomabredemeyeri*), a species described in 1827 based on type material collected in Venezuela during the poorly documented Austrian Märter expedition (1783–1788). *Strychnosbredemeyeri* is an unarmed liana with solitary tendrils and axillary inflorescences that occurs in Neotropical rainforests and savannas in Brazil, Guyana, Trinidad and Tobago, and Venezuela. We clarify here the nomenclatural status of *Lasiostoma* Schreb., an illegitimate and superfluous genus currently in synonymy under *Strychnos*, and its former species *Lasiostomabredemeyeri* [= *Strychnosbredemeyeri*]. Also, we lectotypify *S.pedunculata* and *S.trinitensis*, both taxa currently synonyms of *S.bredemeyeri*.

## ﻿Introduction

*Strychnos* L. is a pantropical genus of lianas, shrubs, or trees with ca. 200 spp., making it the largest genus in Loganiaceae (Gentianales; Struwe et al. 2018). It is well-known for its poisonous properties, including being the source of the toxic alkaloid strychnine (Setubal et al. 2021). Many of the ca. 80 species from the Americas were named and described by naturalists and explorers of the Neotropics of the 18^th^ century and onward, who sought the ingredients of the indigenous dart poison *curare* (Krukoff 1972). This interest resulted in the description of new genera, including *Rouhamon* Aubl. and *Lasiostoma* Schreb., both names now placed in synonymy under *Strychnos* (Krukoff and Monachino 1942), and more than a hundred new species or infraspecific taxa, many of which are currently in synonymy or remain in a doubtful taxonomic status among American *Strychnos* (Krukoff and Monachino 1942; Krukoff 1972; Krukoff 1979a).

*Rouhamon* was published by the French pharmacist and botanist Jean Baptiste C. F. Aublet based only on the type species *R.guianensis* Aubl. (Aublet 1775: 93). *Rouhamonguianensis* [= *Strychnosguianensis* (Aubl.) Mart.] was collected during Aublet’s trip to French Guyana in 1762–1764 and described as being part of the preparation of curare by the indigenous tribe of the Galibis. Schreber (1789: 85) published *Lasiostoma* as an avowed substitute (replacement name) of *Rouhamon* (i.e., the protologue of *Lasiostoma* explicitly cited *R.guianensis* as the only species included in the new genus). Not many botanists followed Schreber, but a few species were eventually published under *Lasiostoma*, including *L.bredemeyeri* Schult. & Schult.f. (1827: 64) [= *Strychnosbredemeyeri* (Schult. & Schult.f.) Sprague & Sandwith], an unarmed liana with solitary tendrils and axillary inflorescences that occurs in Neotropical rainforests and savannas in Brazil, Guyana, Trinidad and Tobago, and Venezuela (Krukoff 1972).

*Lasiostomabredemeyeri* was published based on material collected by Franz Bredemeyer, a gardener who traveled from Austria to Martinique, Puerto Rico, and Venezuela on the poorly documented Franz Joseph Märter expedition during 1783–1788 (von Jacquin 1797; Stafleu 1972). Bredemeyer stayed in Venezuela between 1786–1788 (Lindorf 2004) and the eventual type material of *L.bredemeyeri* was taken back to Austria and distributed to personal herbaria such as those of Carl L. von Willdenow and Nicolaus J. von Jacquin [later incorporated into herbaria in Berlin, Germany (B) and Vienna, Austria (W), respectively]. The protologue of *L.bredemeyeri* presents scarce information about the species, including the name of Willdenow and a short and controversial diagnosis associating the herbarium designation “Lasiostomaglabrum” with the description “*corollis fauce glabris*”.

After the publication of *Lasiostomabredemeyeri*, De Candolle (1845a: 18), Progel (1868: 284), Sprague and Sandwith (1927: 128), and Krukoff and Monachino (1942: 321; 1946: 192) treated *L.bredemeyeri* as a doubtful species, not being able to locate its type material. Krukoff (1965: 50) finally located Bredemeyer’s specimen deposited in Jacquin’s herbarium at W (*Bredemeyer s.n.*, W0078191) due to a suggestion made by another specialist of *Strychnos*, Noel Y. Sandwith, noting that “Franz Bredemeyer was probably a gardener sent out to Venezuela by Jacquin”. The Bredemeyer’s specimen at W was identified by Krukoff as *Strychnospedunculata* (A.DC.) Benth. (1964), based on the pilose inner surface of the corolla tube. Because of the morphological contradiction between the pilose inner surface of the corolla in Bredemeyer’s specimen at W and the diagnosis of *L.bredemeyeri* (mouth of the corolla glabrous), Krukoff kept this material in doubt as the possible lost type specimen of *S.bredemeyeri*. Later, Krukoff and Barneby (1969b: 181) attempted to locate Bredemeyer’s specimen deposited in Willdenow’s herbarium at B (*Bredemeyer s.n.*, BW02865000, BW02865010), but the material was not found at the time probably because Willdenow’s herbarium is still kept separate from the main collection of B since 1943 (B–W), when it was removed in an attempt to avoid being destroyed by bombing during WWII (Hiepko 1987). Therefore, Krukoff and Barneby (1969b) designated Bredemeyer’s specimen at W as the lectotype of *Lasiostomabredemeyeri*, citing *S.bredemeyeri* as the accepted name with priority of use, and placing *S.pedunculata* and *S.trinitensis* Griseb. (an old synonym of *S.pedunculata*; Sandwith 1933: 397) in its synonymy.

*Rouhamonpedunculatum* A.DC. [= *S.pedunculata*] was published in De Candolle (1845b: 561) based on material collected by the brothers Robert and Richard Schomburgk during their expedition to Guyana and vicinities in 1840–1844 (*R.H. Schomburgk 482*, BM [BM000952958], F [V0062158F, V0062159F], G [G00368309, G00368310, G00132188], GH [GH00076757], NY [NY00297387], P [P00647601, P00647602], TCD [TCD0000695], US [US00112974], W [W0078192, W0196054]). *Strychnospedunculata* was cited by the brothers Schomburgk as being part of the preparation of curare by the indigenous tribe of the Macusis (Schomburgk 1848a: 179). The type material of *S.trinitensis* (*H. Crueger s.n.*, GOET [GOET005464], K [K000573430], NY [NY00297487], TRIN [Catalog Nos. 258, 1529], US [US01100481, US00112982]) was collected by Hermann Crueger, a German-born apothecary who settled in Trinidad and Tobago in 1841, becoming a government botanist and director of the Botanical Garden during 1857–1864 (Stafleu and Cowan 1976). This material was distributed to other herbaria by the Trinidad Botanical Garden, and eventually formed the basis for Grisebach’s (1861: 407) new species *S.trinitensis*, which at the time was thought to be endemic to Trinidad.

No further information about the correct nomenclatural status of the genus *Lasiostoma* Schreb., the basionym of *S.bredemeyeri*, and the location of Bredemeyer’s specimen at B was published by Krukoff in his subsequent publications (Krukoff 1972; Krukoff and Barneby 1973, 1974; Krukoff 1976, 1977, 1978, 1979a, 1979b, 1980, 1982a, 1982b). Also, there is no publication updating the location and typification of the type materials of *S.pedunculata* and *S.trinitensis*, both taxa currently in synonymy under *S.bredemeyeri*. Thus, we clarify the nomenclature of *Lasiostoma* and its former species *L.bredemeyeri*, describe the location of the type materials of all the names involved, and lectotypify its synonyms *S.pedunculata* and *S.trinitensis*.

## ﻿Material and methods

We examined herbarium material from 13 herbaria; seen in-person at F, GH, NY, and US; or seen online as digital images from B, BM, G, GOET, K, P, TCD, TRIN, and W (acronyms according to Thiers, updated continuously). All nomenclatural actions follow the Shenzhen Code (Turland et al. 2018).

## ﻿Results and discussion

### ﻿Notes on *Lasiostoma* and *Rouhamon*

Schreber (1789: 85) validly published the monospecific genus *Lasiostoma* as an avowed substitute (replacement name) for *Rouhamon* witnessed by the fact that the only species included in *Lasiostoma* was *Rouhamonguianensis* (Turland et al. 2018: Art. 6.11). However, *Rouhamon* (Aublet 1775: 93) is legitimate and has priority, making *Lasiostoma* an illegitimate and superfluous name (Turland et al. 2018: Art. 52.1, 52.2). Later, Bentham (1843: 224) published the homonym *Lasiostoma* Benth. in Rubiaceae (type species: *L.loranthifolium* Benth.), but this name is also illegitimate because *Lasiostoma* Schreb. had already been described (Turland et al. 2018: Art. 53.1). The illegitimacy of *Lasiostoma* Schreb. does not affect the legitimate status of species effectively published under it, unless the publications of these species are in disagreement with other rules of the Code (Turland et al. 2018: Art. 55.1). *Rouhamon* and *Lasiostoma* are correctly placed as synonyms of *Strychnos*.

### ﻿Notes on *Strychnosbredemeyeri*

**Figure 1.**
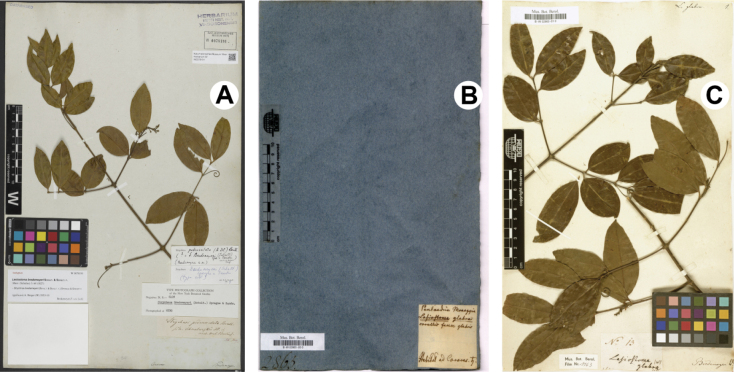
Type specimens of *Lasiostomabredemeyeri* Schult. & Schult.f **A** lectotype conserved in the Jacquin herbarium at W (*Bredemeyer s.n.*; W barcode W0078191) **B, C** isolectotype conserved in the Willdenow herbarium at B (*Bredemeyer s.n.*; B barcodes BW02865000 and BW02865010, respectively).

We found two specimens of the type material of *Lasiostomabredemeyeri*, the lectotype designated by Krukoff and Barneby (1969b: 181) at W (Jacquin’s herbarium; *Bredemeyer s.n.*, W0078191; Fig. 1A) and an isolectotype at B (Willdenow’s herbarium; *Bredemeyer s.n.*, BW02865000, BW02865010; Fig. 1B, C). Both specimens have the names “Bredemeyer” and “Caracas” annotated, but only the isolectotype at B has an original label containing the same diagnosis used in the protologue of the species: “Lasiostomaglabra, *corollis fauce glabris*” (Fig. 1B).

**Figure 2.**
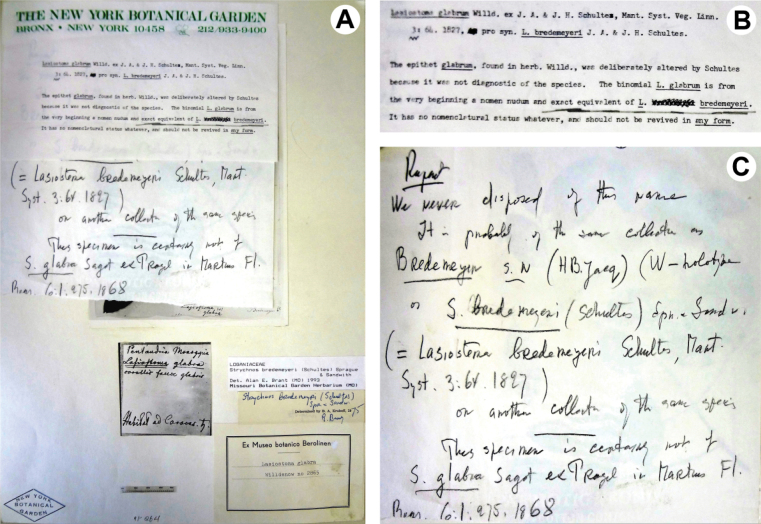
**A–C** sheet deposited in the type photograph collection of the New York Botanical Garden (Negative No. 1163; sheet without barcode or access number and not available online) containing a photograph of the isolectotype of *Lasiostomabredemeyeri* Schult. & Schult.f. conserved in the Willdenow herbarium at B (*Bredemeyer s.n.*; Fig. 1B,C) and two overlapping notes made by Krukoff **A** overview; note the detail of the original label containing the description “Lasiostomaglabra, *corollis fauce glabris*” (at center) and Krukoff’s identification tag made in 1975 (at right) **B** detail of the uppermost typewritten note made by Krukoff **C** detail of the basalmost handwritten note made by Krukoff (all images made by R. B. Setubal).

Krukoff never mentioned Bredemeyer’s specimen at B (BW02865000, BW02865010) in any of his subsequent works (Krukoff 1972; Krukoff and Barneby 1973, 1974; Krukoff 1976, 1977, 1978, 1979a, 1979b, 1980, 1982a, 1982b), but we found a photograph of this specimen housed in the type photograph collection of the New York Botanical Garden (Negative No. 1163; sheet without barcode or access number and not available online; Fig. 2). The photograph was identified as *S.bredemeyeri* by Krukoff in 1975 and contains a typewritten and a handwritten note made by Krukoff (Fig. 2B, C). In these notes, Krukoff stated that the diagnosis “Lasiostomaglabra, *corollis fauce glabris*” (BW02865000; Fig. 1B) was probably proposed by Willdenow, but the epithet ‘glabrum’ was deliberately altered by Schultes and Schultes (1827: 64) to ‘bredemeyeri’ because a glabrous corolla throat was not diagnostic of the species based on the pilose inner surface of the corolla in Bredemeyer’s specimen. The etymology of *Lasiostoma* is about the hairy, woolly (lasio- from Greek) mouth (-stoma from Greek), probably referring to the characteristic whitish-woolly inner surface of the corolla tube of *S.guianensis* that extends from the near base to the lower half of the corolla lobes (Sandwith 1933: 400; Krukoff 1972: 236). Schultes and Schultes (1827: 64) perhaps changed the epithet ‘glabrum’ to ‘bredemeyeri’ to avoid a contradictory combination such as “Lasiostomaglabrum”, however, the contradictory diagnosis was kept the same (glabrous corolla throat). “Lasiostomaglabrum” was cited as part of the diagnosis of *L.bredemeyeri*, and therefore this cannot be considered as a name because the new taxon described was *L.bredemeyeri*. Consequently, “L. glabrum” is not a potential homonym of *Strychnosglabra* Sagot ex Progel (Progel 1868: 275) as also expressed in Krukoff’s notes (Fig. 2B, C). “Lasiostomaglabrum” was eventually cited by De Candolle (1845a: 18), but is a nomen nudum (Turland et al. 2018: Art. 38.1, 38.2, 38.8).

Despite the illegitimacy of *Lasiostoma* Schreb. and considering that a validating description need not be diagnostic of the new taxon (Turland et al. 2018: Art. 38.1), *L.bredemeyeri* was validly published and its nomenclatural application is correct due to priority over the other taxa currently in its synonymy (see below). Sprague and Sandwith (1927: 128) were the first authors to validly publish the new combination of *Strychnosbredemeyeri* (Turland et al. 2018: Art. 41.3) as mostly cited in all subsequent publications of Krukoff, except in Krukoff and Barneby (1969a: 45) when it was incorrectly attributed to Badillo (1947: 247).

### ﻿Notes on *Strychnospedunculatum*

The type material of *Rouhamonpedunculatum* was collected during the brothers Robert (R.H. Schomburgk) and Richard Schomburgk’s (M.R. Schomburgk) expedition to Guyana in 1840–1844. While the protologue of the species mentions the type locality as “In Guyana brit. ad Roraima”, the route indicated on the map and the respective text in Schomburgk (1922: 140; Map. 5) suggests that the type material was collected during the passage through the confluence of the Surumu and Cotingo Rivers, close to “Mount Piriwai” in October 1842, in the State of Roraima, Brazil. The holotype of *R.pedunculatum* was not indicated, and the lectotype selected here (G barcode G00132188) has the indication “Herb. Prodr. (G–DC)”, suggesting it to be part of the original material seen by De Candolle (Turland et al. 2018: Art. 9.3, 9.4). We also verified the existence of a second specimen identified as *R.pedunculatum* housed at G–DC (G barcode G00132189), but this sheet does not have any label or information about this collection. We contacted herbarium G about the origin of this second specimen, but the staff responded that there is not enough information to accurately tell whether these two images containing two different barcodes might be interpreted as two or only one gathering (Fred Stauffer, curator of herbarium G, pers. comm). Therefore, we did not include this specimen in the type material of *R.pedunculatum*.

**Figure 3.**
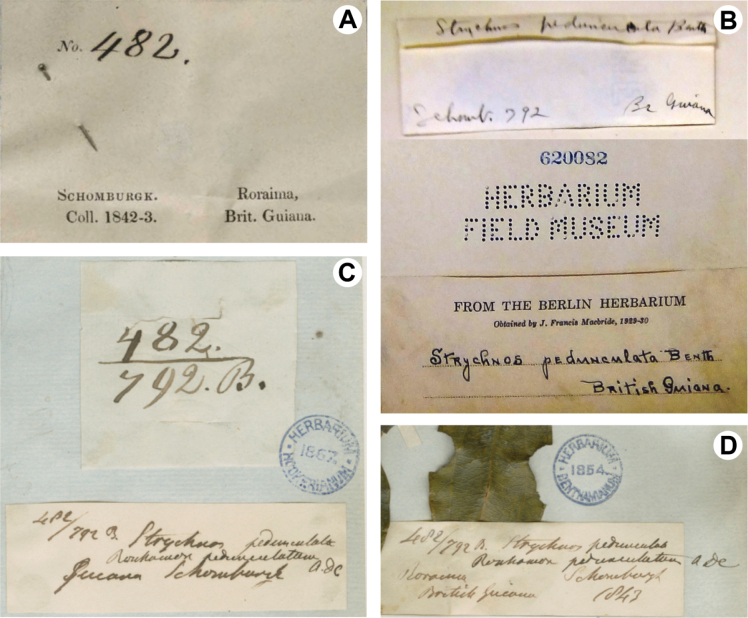
Comparison of labels on four specimens of *Rouhamonpedunculatum* A.DC **A** lectotype showing original label “*Schomburgk 482*” (G barcode G00132188) **B–D** specimens of *R.pedunculatum* without original labels and containing alternative numbers **B** specimen with the alternative number “792” (F Catalog No. 620082; not available online; image from R. B. Setubal) **C, D** specimens with the numbers “482” and “792.B” written juxtaposed (K barcodes K000573484 and K000573485, respectively).

Three specimens without original labels but bearing written indications of “Schomburgk” and “Br. Guiana” have the alternative collector number “792” (photograph of specimen at F, Catalog No. 620082; not available online – not to be confused with F barcode V0044336F; see further) or “792.B” (K barcodes K000573484 and K000573485; Fig. 3). This number led to different citations of the type material of *R.pedunculatum* by different authors: “*R.H. Schomburgk 482* and *792B*” (Bentham 1857: 105; Progel 1868: 275), “*R.H. Schomburgk 482=792B*” (Sandwith 1933: 397, 1935: t. 3225), and “*R.H. Schomburgk 482* and *M.R. Schomburgk 792B*” (Krukoff and Monachino 1942: 291). The F specimen with only the number “792” (Fig. 3B) consists of a single fragment taken from an original specimen housed at B that unfortunately appears not to have been photographed by J. Francis McBride of the Field Museum and was probably destroyed during WWII. While this specimen is not available online, we verified the existence of a second collection labeled as “R.H. Schomburgk 792”, available online at F, but this is not a specimen of *Strychnos*, and is identified as type material of *Lecythisschomburgkii* O.Berg (F barcode V0044336F).

The two K specimens (Fig. 3C, D) are the only material that have both numbers (“482” and “792.B”) written juxtaposed, and a possibility is that the letter “B” was added to the number “792” to represent the existence of the collection number “792” written in the label of the B specimen destroyed in WWII. Due to the lack of evidence to confirm if these three specimens represent true duplicates of “*R.H. Schomburgk 482*”, we excluded these specimens from the type material of *R.pedunculatum*, at least until new evidence is available.

We also examined two additional specimens labeled *M.R. Schomburgk s.n.* (BR barcode BR0000005859795; GH, sheet without barcode or access number and not available online) without date, locality or collection number, and with label and handwriting that clearly differs from all other specimens. This material was cited as additional material examined and not type collection by Progel (1868: 275) and Sandwith (1933: 397), a position that we also share. Finally, the specimens BR0000005859795 and W0078192 also have the designation “*Strychnosschomburgkiana* Klotzsch” (Schomburgk 1848b: 1144), which is a *nomen nudum* (Turland et al. 2018: Art. 38.1, 38.2, 38.8).

### ﻿Notes on *Strychnostrinitensis*

**Figure 4.**
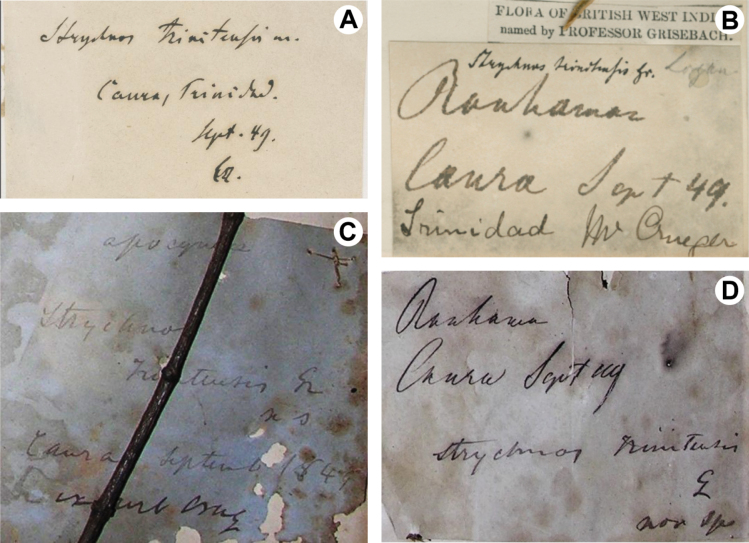
Comparison of labels on four type specimens of *Strychnostrinitensis* Griseb **A** lectotype (*H. Crueger s.n.*, GOET barcode GOET005464) **B–D** isolectotypes **B** K barcode K000573430**C** TRIN Catalog No. 258**D** TRIN Catalog No. 1529.

The protologue of *S.trinitensis* notes “Trinidad, *Crueger* at Caura”, indicating that the type material was collected by Herman Crueger (past director of the Trinidad Botanical Garden) at the Caura or Tacarigua River, a tributary of the Caroni River in the Northern Range of Trinidad Island. All the type material examined has the information “Caura, Sept. 1849”, and the name “Crueger” written with different legibility (Fig. 4). The protologue of *S.trinitensis* did not indicate the holotype, and the lectotype selected here (GOET barcode GOET005464) has the stamp of “Herbarium Grisebachianum”, suggesting that it is part of the original material seen by Grisebach (1861: 407; Turland et al. 2018: Art. 9.3, 9.4).

***Strychnosbredemeyeri* (Schult. & Schult.f.) Sprague & Sandwith, Bull. Misc. Inform. Kew 3: 128 . 1927. Type: Venezuela. Caracas, [1786–1788] (fl.), *F. Bredemeyer s.n.* Lectotype (designated by Krukoff & Barneby [1969b: 181]): W [W0078191] ; isolectotype: B [BW02865000 , BW02865010] .**

≡ *Lasiostomabredemeyeri* Schult. & Schult.f., Mant. 3: 64. 1827.

≡ *Rouhamonbredemeyeri* (Schult. & Schult.f.) DC. in A.P. De Candolle, Prodr. 9: 18. 1845.

≡ *Lasiostomaglabrum* Willd. ex DC. in A.P. De Candolle, Prodr. 9: 18. 1845. nom. nud.

= *Rouhamonpedunculatum* A.DC. in A.P. De Candolle Prodr. 9: 561. 1845. Type: British Guiana [Brazil]. [Roraima]: ad Roraima, [Oct] 1842 (fl.), *R.H. Schomburgk 482*. Lectotype (designated here): G [G00132188]; isolectotypes: BM [BM000952958], F [V0062158F, V0062159F], G [G00368309, G00368310], GH [GH00076757], NY [NY00297387], P [P00647601, P00647602], TCD [TCD0000695], US [US00112974], W [W0078192, W0196054]).

≡ *Strychnosschomburgkiana* Klotzsch, Reis. Br.-Guiana [Ri. Schomburgk] 3: 1144. 1848. nom. nud.

≡ *Strychnospedunculata* (A.DC.) Benth., J. Proc. Linn. Soc., Bot., 1: 105. 1857.

= *Strychnostrinitensis* Griseb., Fl. Brit. W.I.: 407. 1861. Type: Trinidad and Tobago. Caura, Sep 1849 (fl.), *H. Crueger s.n.* Lectotype (designated here): GOET [GOET005464]; isolectotypes: K [K000573430], NY [NY00297487], TRIN [Catalog Nos. 258, 1529], US [US01100481, US00112982]).

## ﻿Additional information

### Author ORCIDs

Robberson Bernal Setubal https://orcid.org/0009-0006-1677-9137

Lena Struwe https://orcid.org/0000-0001-6074-5758

Jefferson Prado https://orcid.org/0000-0003-4783-3125

Rafaela Campostrini Forzza https://orcid.org/0000-0002-7035-9313

### Data availability

All of the data that support the findings of this study are available in the main text.
